# WWP2 drives the progression of gastric cancer by facilitating the ubiquitination and degradation of LATS1 protein

**DOI:** 10.1186/s12964-023-01050-2

**Published:** 2023-02-17

**Authors:** Jianping Zou, Ling Zhou, Yi Le, Zhi Fang, Min Zhong, Fengting Nie, Xianpin Wei, Xiaomei Zhang, Zhen Chen, Lingling Cai, Heng Wang, Jianping Xiong, Ziling Fang, Xiaojun Xiang

**Affiliations:** 1 Department of Oncology, the First Affiliated Hospital of Nanchang University, 1519 Dongyue Avenue, Nanchang, Jiangxi People’s Republic of China; 2 Department of Jiangxi Key Laboratory for Individualized Cancer Therapy, 17 Yongwai Street, Nanchang, 330006 Jiangxi China; 3 Department of Orthopedics, the First Affiliated Hospital of Nanchang University, 1519 Dongyue Avenue, Nanchang, Jiangxi People’s Republic of China

**Keywords:** Gastric cancer, LATS1, WWP2, Progression, Ubiquitination

## Abstract

**Background:**

Large tumor suppressor kinase 1 (LATS1), one of the predominant components of the Hippo pathway, has been characterized as a key player controlling the proliferation and invasion of cancer cells, including gastric cancer (GC) cells. However, the mechanism by which the functional stability of LATS1 is modulated has yet to be elucidated.

**Methods:**

Online prediction tools, immunohistochemistry and western blotting assays were used to explore the expression of WW domain-containing E3 ubiquitin ligase 2 (WWP2) in GC cells and tissues. Gain- and loss-of-function assays, as well as rescue experiments were performed to determine the role of the WWP2-LATS1 axis in cell proliferation and invasion. Additionally, the mechanisms involving WWP2 and LATS1 were assessed by coimmunoprecipitation (Co-IP), immunofluorescence, cycloheximide and in vivo ubiquitination assays.

**Results:**

Our results demonstrate a specific interaction between LATS1 and WWP2. WWP2 was markedly upregulated and correlated with disease progression and a poor prognosis in GC patients. Moreover, ectopic WWP2 expression facilitated the proliferation, migration and invasion of GC cells. Mechanistically, WWP2 interacts with LATS1, resulting in its ubiquitination and subsequent degradation, leading to increased transcriptional activity of YAP1. Importantly, LATS1 depletion abolished the suppressive effects of WWP2 knockdown on GC cells. Furthermore, WWP2 silencing attenuated tumor growth by regulating the Hippo-YAP1 pathway in vivo.

**Conclusions:**

Our results define the WWP2-LATS1 axis as a critical regulatory mechanism of the Hippo-YAP1 pathway that promotes GC development and progression.

**Video Abstract**

**Supplementary Information:**

The online version contains supplementary material available at 10.1186/s12964-023-01050-2.

## Introduction

Gastric cancer (GC), one of the most common malignancies, is the fourth leading cancer deaths worldwide [1]. Despite many preventions and treatment efforts in recent decades, the incidence and mortality rates of GC are still high due to distant metastasis at the time of diagnosis [2, 3]. However, the precise mechanisms underlying the tumorigenesis and progression of GC remain largely uninvestigated. Therefore, it is urgent to perform a comprehensive analysis of novel therapeutic targets for GC biology research and treatment.

Numerous studies have demonstrated that the Hippo signaling pathway plays an important role in both normal tissue regeneration and tumorigenesis [4, 5]. The core of the Hippo signaling pathway consists of the kinase cascade: MST1/2 and LATS1/2 and the transcriptional coactivator YAP/TAZ [6, 7]. Upon upstream kinase stimulation, LATS1/2 phosphorylates YAP1, resulting in its retention in the cytoplasm and its inactivation. Otherwise, unphosphorylated YAP1 enters the nucleus and binds to transcription factors such as TEAD, leading to the activation of downstream targets, such as connective tissue growth factor (CTGF) and cysteine-rich angiogenic inducer 61 (CYR61), which in turn promotes tumorigenesis [8, 9]. Accumulating studies have shown that LATS1 serves as a tumor suppressor [10, 11]; however, the mechanism by which LATS1 stability is regulated remains elusive.

WW domain containing E3 Ub-protein ligase 2 (WWP2), as one of the NEDD4 family of E3 ligase proteins, contains an N-terminal membrane targeting the C2 domain, four internal double tryptophan (WW) domains and a C-terminal HECT domain that confers E3 ligase activity [12]. It is reported to be responsible for a series of physiological processes, such as cell proliferation, transcription regulation and immune responses [12–15]. Previous studies have indicated that WWP2 is abnormally expressed in a variety of solid tumors, such as lung cancer, glioma and liver cancer [16–19]. Evidence suggests that WWP2 interacts with a series of signaling pathways highly related to tumorigenesis, such as the PTEN/PI3K/Akt signaling pathway, Notch3 signaling pathway and TGF-β signaling pathway[20–22]. Although a recent study mentioned a correlation between WWP2 and tumorigenicity in GC [23], the mechanism by which WWP2 exerts its oncogenic functions in GC remains unclear.

In the present study, we found that WWP2 plays an important role in GC tumorigenesis and progression partially in a LATS1-dependent manner by modulating the Hippo-YAP1 signaling pathway. WWP2 upregulation promoted cell growth, migration and invasion in GC cells, while WWP2 depletion resulted in the opposite effects. Mechanistically, WWP2 directly targets the LATS1 protein for ubiquitination and degradation, further inhibiting the Hippo-YAP1 pathway. In conclusion, our study demonstrates a critical role of the WWP2-mediated Hippo-YAP1 pathway in the progression of GC and provides novel insights into therapeutic targets for GC patients.

## Materials and methods

### Tissue specimens and ethics statement

A total of 130 paraffin-embedded gastric cancer specimens were obtained from treatment-naïve patients who underwent surgical resection at the First Affiliated Hospital of Nanchang University between 2013 and 2016. Eight fresh primary GC tissues and their matched normal gastric tissues were immediately frozen at − 80 °C upon radical resection in the Department of Surgery at our hospital. The clinicopathological features of the GC patients are provided in Table 1 and Additional file 1: Table S1. This study was approved by the corresponding ethics committee and in accordance with the Declaration of Helsinki. Informed written consent was acquired from all patients.

**Table 1:** Association of WWP2 levels with clinical and pathological features of GC patients

Variables	*N*	Tumor WWP2 expression		*P* value
		WWP2^High^ (%)	WWP2^Low^ (%)	
Age (years)				
< 65	66	38(57.6)	28(42.4)	0.567
≥ 65	64	40(62.5)	24(37.5)	
Gender				
Male	75	43(57.3)	32(42.7)	0.469
Female	55	35(63.6)	20(36.4)	
Tumor size (cm)				
< 5	51	28(54.9)	23(45.1)	0.340
≥ 5	79	50(63.3)	29(36.7)	
Differentiation				
Well or Moderately	68	37(54.4)	31(45.6)	0.173
Poor	62	41(66.1)	21(33.9)	
TNM stage				
I-II	57	27(47.4)	30(52.6)	**0.009**
III-IV	73	51(69.9)	22(30.1)	
Depth of invasion				
T1-T2	54	25(46.3)	29(53.7)	**0.007**
T3-T4	76	53(69.7)	23(30.3)	
Tumor location				
Proximal	59	34(57.6)	25(42.4)	0.615
Distal	71	44(62.0)	27(38.0)	
Lauren classification				
Intestinal type	65	35(53.8)	30(46.2)	0.152
Diffuse type	65	43(66.2)	22(33.8)	
Lymph node metastasis				
N0	60	30(50.0)	30(50.0)	**0.031**
Nx	70	48(68.6)	22(31.4)	

*P*-values determined using *χ*2 test

Bold: *P* ≤ 0.05 were regarded as statistically significant

### Cell culture and transfection

The human gastric epithelial cell line GES-1 and GC cell lines (AGS, BGC-823, MGC-803, SGC-7901, HGC-27 and MKN-45) were acquired from the Shanghai Institute of Cell Biology, China Academy of Sciences. The GC cells were maintained in RPMI-1640 medium or DMEM (Dulbecco’s modified Eagle’s medium, HyClone, Logan, UT, USA) supplemented with 10% fetal bovine serum (FBS; BI, Kibbutz, Israel) in an atmosphere with 5% CO_2_ at 37 °C. In addition, all cell samples were authenticated by short tandem repeat (STR) sequencing and tested for mycoplasma detection.

The WWP2 shRNAs, LATS1 siRNAs, HA-WWP2 and Flag-LATS1 plasmids were designed and purchased from GenePharma (Shanghai, China). The sequences of shRNAs and siRNAs used in our study are provided in Additional file 2: Table S2. GC cells were plated evenly the day before transfection and transfected with the indicated treatments using TurboFect transfection reagent (Thermo Scientific, R0532, Waltham, USA) for 48 h. In addition, WWP2 shRNA lentivirus and relevant control lentivirus were constructed by Jikai Gene Chemical Technology Company (Shanghai, China). As mentioned previously [24], lentivirus production in infected GC cells was conducted according to the manufacturer’s instructions, and puromycin (2 μg/ml) was used to establish the stable cell lines.

### Western blotting analysis

Prior to western blotting experiments, GC cells, ground human GC tissue samples and xenograft tumors were lysed with 4 °C precooled RIPA buffer. The total proteins were separated by 10% SDS-PAGE gels and then transferred to nitrocellulose membranes (Millipore, MA, USA). After blocking with 5% skim milk powder, the membranes were incubated with primary antibodies at 4 °C overnight, including anti-WWP2 (1:2000, #12,197–1-AP, Proteintech, Wuhan, China), anti-LATS1 (1:1000; #3477; Cell Signaling Technology, Danvers, USA), anti-YAP1 (1:1000; #14,074; Cell Signaling Technology, Danvers, USA), anti-phospho-YAP (1:1000; #13,008; Cell Signaling Technology, Danvers, USA), anti-CTGF (1:1000; #86,641; Cell Signaling Technology, Danvers, USA), anti-CYR61 (1:1000; #14,479; Cell Signaling Technology, Danvers, USA), anti-HA (1:1000; #ab9110; Abcam, Cambridge, USA), anti-Flag (1:1000; #A2220; Sigma‒Aldrich, USA), anti-β-actin (1:1000; #AF7018; Affinity, Jiangsu, China) and anti-GAPDH (1:1000; #60,004–1-Ig; ProteinTech, Wuhan, China). The next day, each membrane was washed three times with Tris-buffered saline Tween (TBST, T1085, Solarbio, Beijing, China) and incubated with secondary antibodies at room temperature for 1 h. Then, the target proteins were detected using ECL reagent (Millipore, MA, USA).

### Coimmunoprecipitation (Co-IP) assays

To investigate the interaction of HA-WWP2 and Flag-LATS1 exogenously, 800 μg of total protein lysed from GC cells was incubated with anti-Flag beads (#M185-11 MBL, Tokyo, Japan) or anti-HA beads (#M180-11 MBL, Tokyo, Japan) at 4 °C for 2 h. For the endogenous binding assays, 600 μg of cell lysates were incubated with anti-LATS1 antibody or control IgG at 4 °C overnight. The next day, 40 μL Protein A/G agarose (Santa Cruz Biotechnology, Santa Cruz, CA, USA) was added, and the lysates were rotated for 1 h. The beads were washed with lysis buffer at least three times and denatured with 2 × SDS-PAGE protein loading buffer (P1325, Solarbio, Beijing, China). Then, the immunoprecipitated proteins were detected by western blotting analysis using anti-Flag (T0003, Affinity Biosciences, Changzhou, China) or anti-HA (T0008, Affinity Biosciences, Changzhou, China) primary antibodies.

### Immunohistochemistry (IHC) assays

Immunohistochemical staining assays were performed as previously described [25]. The IHC results were blindly evaluated by two pathologists on the basis of the score index (SI) scores (multiplying the percentage of positively stained cells and the intensity of staining). The percentage was scored as 0 (0%), 1 (0–25%), 2 (25–50%), 3 (50–75%) and 4 (75–100%), and the intensity of staining was scored as 0 (negative), 1 (weak), 2 (moderate) and 3 (strong). Samples with SI score < 6 were considered to have low WWP2 expression, and the rest were considered to have high WWP2 expression.

### RNA extraction and quantitative real-time PCR (qRT‒PCR) assays

Total RNA isolated from GC cells and xenograft tissues was extracted using TRIzol reagent (Invitrogen; Thermo Fisher Scientific, CA, USA) as previously described [26]. cDNA was obtained from total RNA using a reverse transcription kit (TransGen Biotech, Beijing, China). Quantitative real-time PCR assays were conducted using SYBR Green Mix (Bio-Rad, Hercules, CA, USA) according to the manufacturer’s protocol. The primer sequences utilized in our study are listed in Table 2, and GAPDH served as an internal loading control. The relative mRNA expression of the indicated genes was assessed by the 2^−ΔΔCt^ method.

**Table 2:** Primer sequences used in qRT-PCR assays

Gene	Primer sequences
WWP2	Forward: 5’-AGGCTAAAGAGGGCTGGAGT-3’
	Reverse: 5’-GCTTTGCGGACACCACTTTC-3’
YAP	Forward: 5’-TCGTTTTGCCATGAACCAGA-3’
	Reverse: 5’-GGCTGCTTCACTGGAGCACT-3’
LATS1	Forward: 5’-TGGTGTTAAGGGGAGAGCCA-3′
	Reverse: 5′-CAGTCCAAACCTCTGGACCCT-3′
CYR61	Forward: 5’-CAGGACTGTGAAGATGCGGT-3’
	Reverse: 5’-GCCTGTAGAAGGGAAACGCT-3’
AREG	Forward: 5’-TGTCGCTCTTGATACTCGGC-3’
	Reverse: 5’-ATGGTTCACGCTTCCCAGAG-3’
CTGF	Forward: 5′-CCTGGTCCAGACCACAGAGT-3′
	Reverse: 5′-TGGAGATTTTGGGAGTACGG-3′
GAPDH	Forward: 5’-CACCCACTCCTCCACCTTTG-3’
	Reverse: 5’-CCACCACCCTGTTGCTGTAG-3’

### Cell counting kit-8 (CCK-8) and colony formation assays

The CCK-8 assay (Beyotime, Shanghai, China) was performed to evaluate cell growth ability according to the manufacturer’s instructions. The transfected cells (1.0 × 10^3^/well) were seeded into 96-well plates, and 10 μL CCK-8 reagent was added at the indicated time each day for 5 days. After incubation for 1 h, the absorbance of the samples was measured using a microplate reader (SpectraMax M5e, CA, USA) at 450 nm to assess cell viability by drawing growth curves.

Equal numbers (0.8 × 10^3^/well) of transfected GC cells were plated into 6-well plates, and the medium was changed every 4 days until the colonies were visible to the naked eyes after approximately 2 weeks. Then, the cells were fixed with 4% paraformaldehyde (PFA) and stained with 1% crystal violet at room temperature for 20 min. The colonies were counted by ImageJ software. All biological experiments were conducted at least three times.

### Wound healing and transwell assays

The transfected GC cells were seeded into 6-well plates and cultured until absolutely confluent. Then, a straight line was scratched using a 10 μL sterile pipette tip to make a wound, and the wound was washed carefully with phosphate-buffered saline (PBS). The cells were cultured in serum-free medium, and images were taken every 12 h to evaluate wound healing.

Bovine serum albumin (BSA) solution (10 g/L) was used to dilute the liquefied Matrigel gel at a proportion of 8:1. Then, 60 μL of the above liquid was added to the bottom of the Transwell chamber. After 30 min, 50 μL BSA solution was added into the chamber to hydrate the Matrigel gel. Then, the transfected cells were plated into the upper chamber and cultured in serum-free medium. In addition, the bottom chamber was filled with complete medium. After incubation for 48 h, the migrated cells were fixed with 4% PFA and stained with 1% crystal violet at room temperature for 20 min. Five random views were selected to take pictures and count the cells. Experiments were performed in triplicate.

### Immunofluorescence staining assays

GC cells transfected with HA-WWP2 and Flag-LATS1 were fixed in 4% PFA for 15 min and then permeabilized with 0.5% Triton X-100 for 10 min. The cells were blocked with 5% bovine serum albumin (BSA) at room temperature for 60 min and subsequently incubated with the anti-HA or anti-Flag antibody at 4 °C overnight. On the second day, the cells were incubated with fluorochrome-conjugated secondary antibodies at room temperature for 45 min protected from light. 4’-6-Diamidino-2-phenylindole (DAPI) was utilized to stain the nucleus for 15 min. Confocal microscopy (Nikon, ECLIPSE Ti2, Tokyo, Japan) was used to observe the cellular localization of WWP2 or LATS1.

### Cycloheximide assays and in vivo ubiquitination analysis

Cycloheximide analysis (CHX, 25 μg/mL) was used to treat the indicated cells transfected with WWP2 shRNA, plasmid, or their corresponding controls, and the cells were harvested at the indicated set times for western blotting analysis. For the ubiquitination assay, GC cells were seeded into a 60 mm dish and transfected with Flag-LATS1, His-Ub, HA-WWP2 or WWP2-shRNA. Thirty-six to 48 h later, cells were treated with MG132 (10 μM) for 6 h to inhibit proteasomal degradation before extraction for further experiments. The cell lysate was then immunoprecipitated with Ni–NTA beads. Western blotting was performed to detect the ubiquitination levels of LATS1 protein.

### Animal models

Female BALB/c mice (aged 5 weeks) were purchased from the Shanghai Lab Animal Research Center (Shanghai, China). BGC-823 cells (4 × 10^6^) infected with scramble shRNA or WWP2 shRNA lentivirus were subcutaneously injected into each mouse. The formula tumor volume = (length × width^2^)/2 was used to calculate the tumor size every 3 days. At 28 days after inoculation, the mice were sacrificed, and xenograft tumors were removed, weighed and photographed. The Ethics Committee of the First Affiliated Hospital of Nanchang University approved the animal studies.

### Statistical analysis

The statistical analysis was conducted by SPSS 20.0 software (Chicago, IL, USA). Student’s two-tailed t test or one-way analysis of variance (ANOVA) was performed to determine the mean difference among groups, and the chi-square test was used to analyze the differences in clinicopathological features. Survival curves were generated via the Kaplan–Meier method and analyzed by the log-rank test. *P* < 0.05 was considered to indicate statistical significance. All experiments were independently performed no less than 3 times. All the data were presented as the mean ± SEM.

## Results

### WWP2 interacts with LATS1

**Fig. 1:**
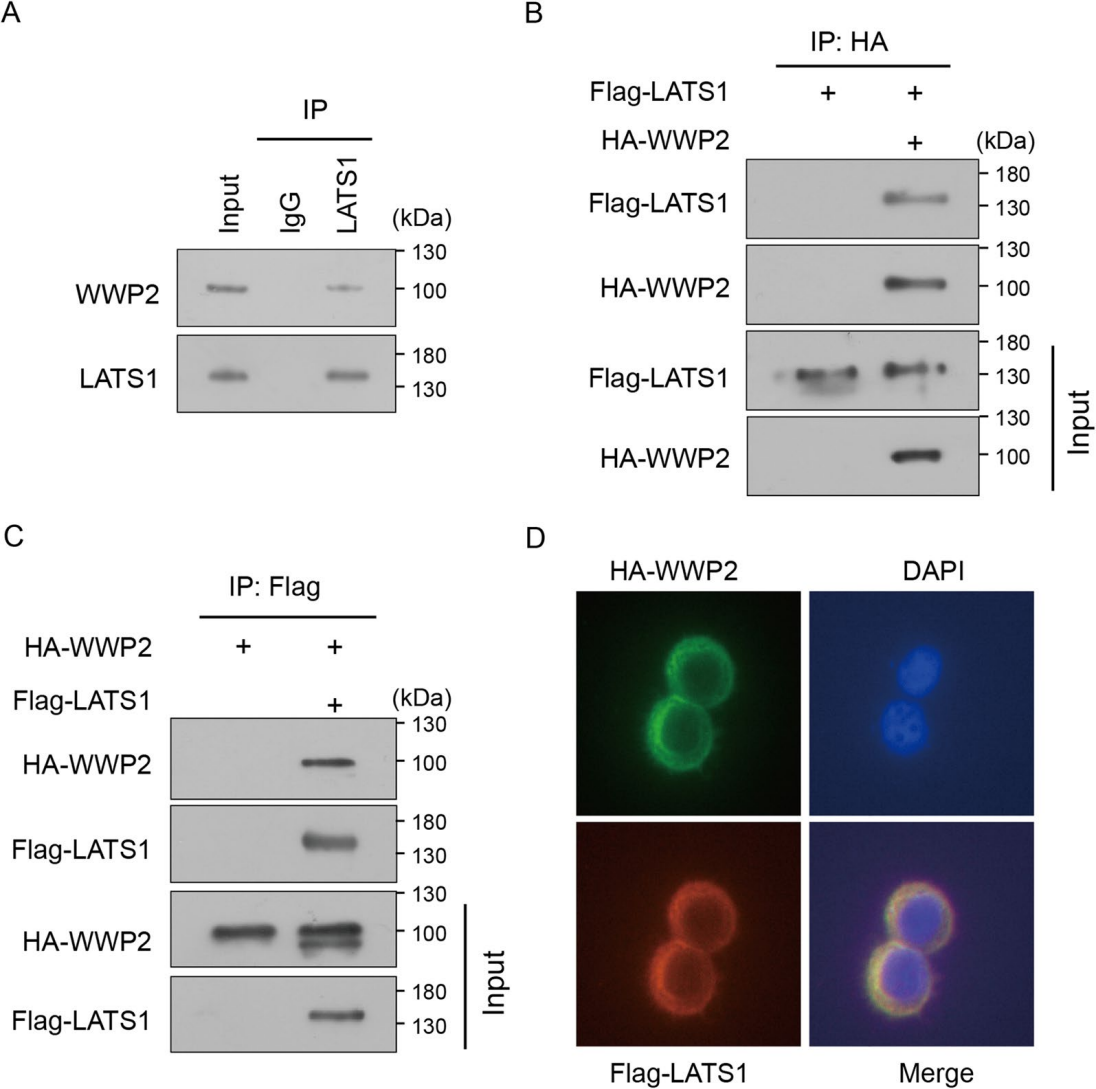
WWP2 interacts with LATS1. **A** The interaction between endogenous WWP2 and LATS1. **B** SGC-7901 cells were transfected with HA-WWP2 alone or together with Flag-LATS1, and the cell lysates were immunoprecipitated with HA-tagged beads and then analyzed by western blotting assays. **C** BGC-823 cells were transfected with Flag-LATS1 alone or together with HA-WWP2, and the cell lysates were immunoprecipitated with Flag-tagged beads and then analyzed by western blotting assays. **D** Immunofluorescence staining for the cellular localization of HA-WWP2 (green) or Flag-LATS1 (red) in SGC-7901 cells. Nuclei were stained with DAPI (blue) (magnification, × 400)

Previous studies have shown that WWP1 promotes the proliferation of breast cancer cells by binding and ubiquitinating the LATS1 protein [27]. WWP2, another member of the NEDD4 family, possessing similar functional domains with WWP1, was reported to be markedly correlated with tumorigenesis and progression in several cancers [16, 19]. However, the association between WWP2 and LATS1 in GC tumorigenesis is unclear. In this study, we confirmed that endogenous WWP2 could directly interact with LATS1 (Fig. 1A) by Co-IP experiments. Moreover, we co-transfected exogenous HA-WWP2 and Flag-LATS1 plasmids into GC cells. The results showed that WWP2 and LATS1 could also interact with each other exogenously (Fig. 1B–C). More importantly, immunofluorescence staining assays also verified that WWP2 and LATS1 were colocalized in the cytoplasm of GC cells (Fig. 1D). Collectively, the above data demonstrate that WWP2 serves as a novel binding partner of LATS1 in GC cells.

### High WWP2 expression is correlated with aggressive clinical characteristics and a poor prognosis in GC patients

**Fig. 2:**
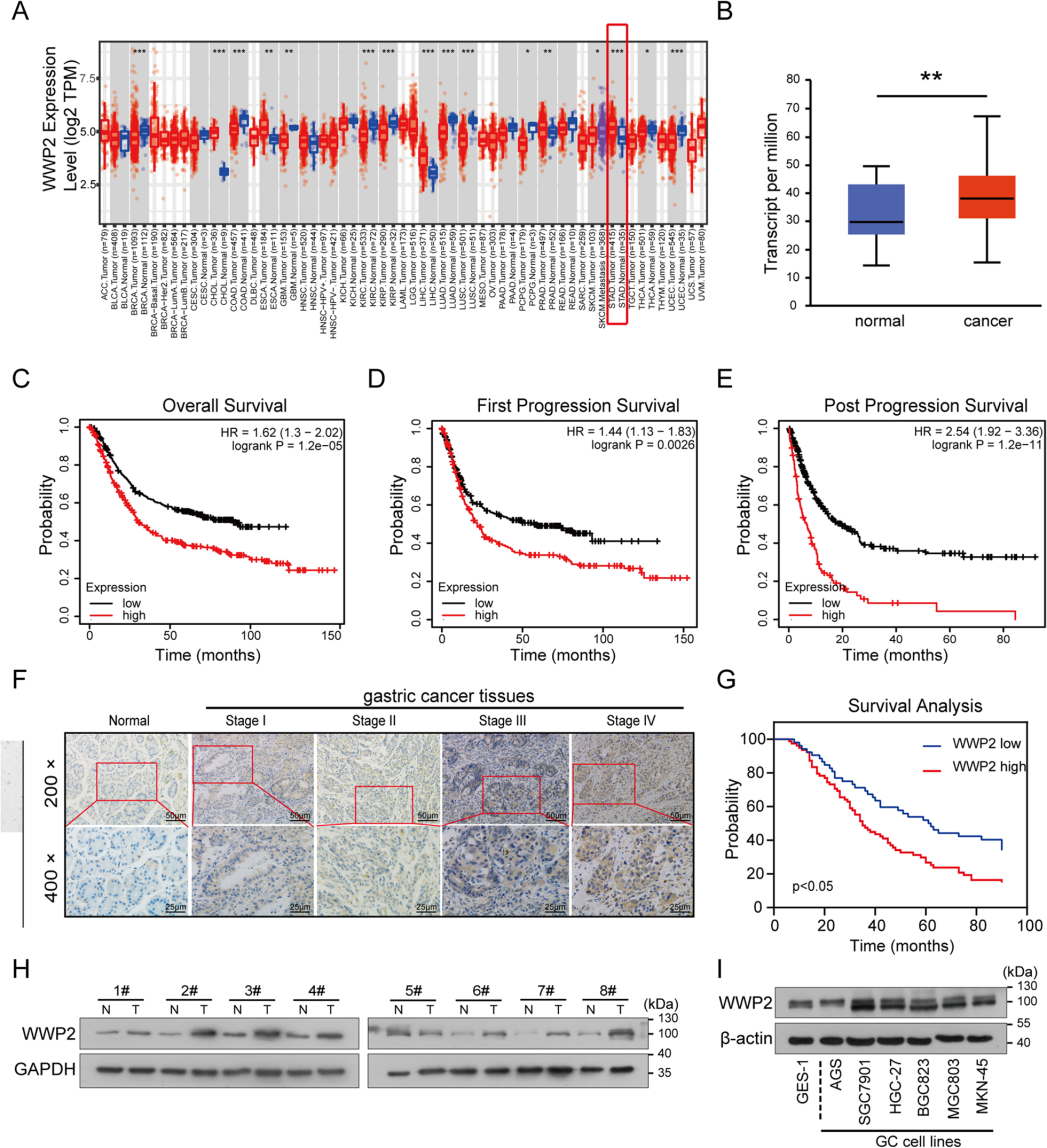
High WWP2 expression is correlated with aggressive clinical characteristics and a poor prognosis in GC patients. **A** The expression levels of WWP2 in different types of tumor tissues and corresponding normal tissues analyzed based on TCGA data obtained from the TIMER (https://cistrome.shinyapps.io/timer/) database (Student’s *t* test, *p* < 0.05). **B** The expression of WWP2 mRNA in GC tissues and matched adjacent nontumor tissues analyzed utilizing the UALCAN (http://ualcan.path.uab.edu/index.html) database (Student’s *t* test, *p* < 0.01). **C**–**E** The association between WWP2 expression and prognosis, including overall survival (OS), time to first progression (TFP)and post progression survival (PPS), in patients with GC obtained from the KM-Plotter (http://kmplot.com/analysis/) database (log-rank test, *p* < 0.05). **F** Representative images of IHC staining of WWP2 protein in human GC tissues and adjacent nontumor tissues. **G** Kaplan‒Meier survival analysis of patients with higher WWP2 expression levels (*n* = 78) and lower WWP2 expression (*n* = 52) (log-rank test, *p* < 0.05). **H** Western blotting analysis was performed to detect the protein expression levels of WWP2 in 8 pairs of GC tissues and paired normal tissues. **I** Western blotting analysis was performed to detect the protein expression of WWP2 in GES-1 and six human GC cell lines (AGS, SGC-7901, HGC-27, BGC-823, MGC-803 and MKN-45). **p < 0.01

To better understand how WWP2 contributes to cancer progression, we first utilized the TIMER database (https://cistrome.shinyapps.io/timer/) to analyze the expression levels of WWP2 in different types of tumor tissues and normal tissues. We found that WWP2 expression was markedly increased in a variety of malignancies, including GC (Fig. 2A). Consistently, the results from the UALCAN database (http://ualcan.path.uab.edu/index.html) revealed that the WWP2 mRNA levels were significantly higher in gastric cancer tissues than in adjacent normal tissues (Fig. 2B , *p*< 0.01). Additionally, Kaplan–Meier Plotter (http://kmplot.com/analysis/) analysis indicated that patients with high WWP2 mRNA expression had worse overall survival (OS), first progression survival (FPS) and post progression survival (PPS) than those with low WWP2 expression (Fig. 2C–E, Affymetrix ID: 1,552,737 _ s_ at).

To further clarify the potential clinical significance and prognostic relevance of WWP2 in GC, immunohistochemistry (IHC) staining was conducted on 130 GC patient specimens. As shown in Fig. 2F and Table 1, WWP2 was significantly upregulated in GC tissues (78/130, 60%), and the staining of WWP2 was markedly enhanced with increasing clinical TNM stage, indicating that WWP2 overexpression contributes to GC tumorigenesis and progression. Consistently, WWP2 upregulation was dramatically associated with TNM stage (*p* = 0.009), lymphatic metastasis status (*P* = 0.031) and depth of tumor invasion (*p* = 0.007) in GC patients (illustrated in Table 1). In addition, Kaplan–Meier survival analysis showed that patients with high WWP2 levels had a significantly lower survival probability than those with low WWP2 expression (*p* < 0.05, Fig. 2G). Similarly, the expression of WWP2 protein in fresh GC tissues was frequently higher than that in adjacent nontumor tissues (Fig. 2H and Additional file 3: Fig. S1A, 7/8, 87.5%). Furthermore, WWP2 also exhibited higher expression in GC cell lines than in GES-1 cells (Fig. 2I and Additional file 3: Fig. S1B). Thus, our data illustrate that WWP2 is highly expressed in GC and correlated with disease progression.

### Modulation of WWP2 expression influenced the malignant behaviors of GC cells in vitro

**Fig. 3:**
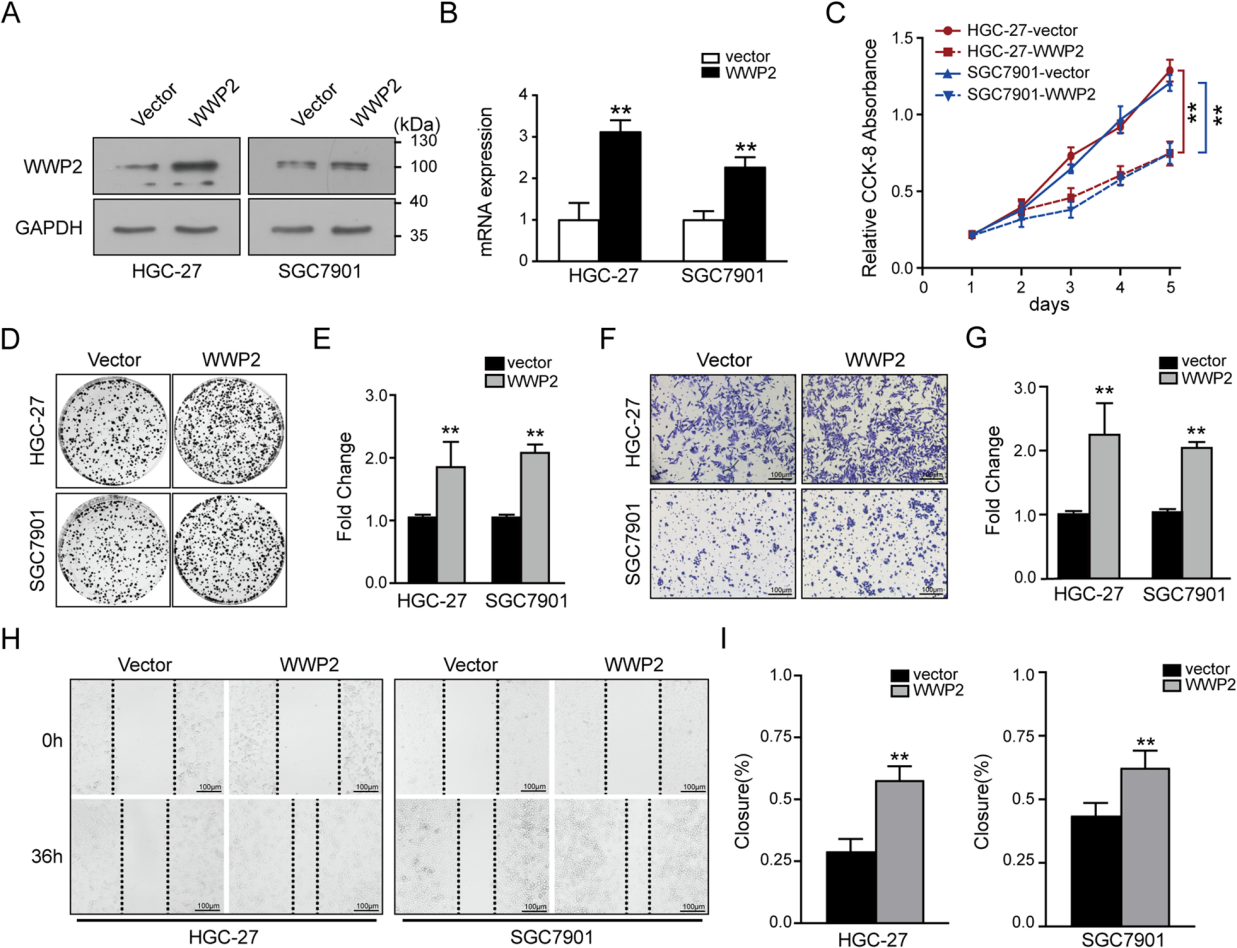
WWP2 overexpression triggers the proliferation, migration and invasion of GC cells. **A, B** The transfection efficiency of the WWP2 expression plasmid was detected by western blotting and qRT‒PCR assays in HGC-27 and SGC-7901 cells. **C** CCK-8 experiments were performed to analyze cell viability. **D, E** Colony formation experiments to analyze the effects of overexpression of WWP2 on colony formation ability. **F, G** Transwell invasion assays of gastric cancer cells transfected with HA-WWP2 or vector. **H, I** Wound healing assays were performed to evaluate the migration abilities in GC cells upon WWP2 upregulation. Student’s t test: *p < 0.05, **p < 0.01

**Fig. 4:**
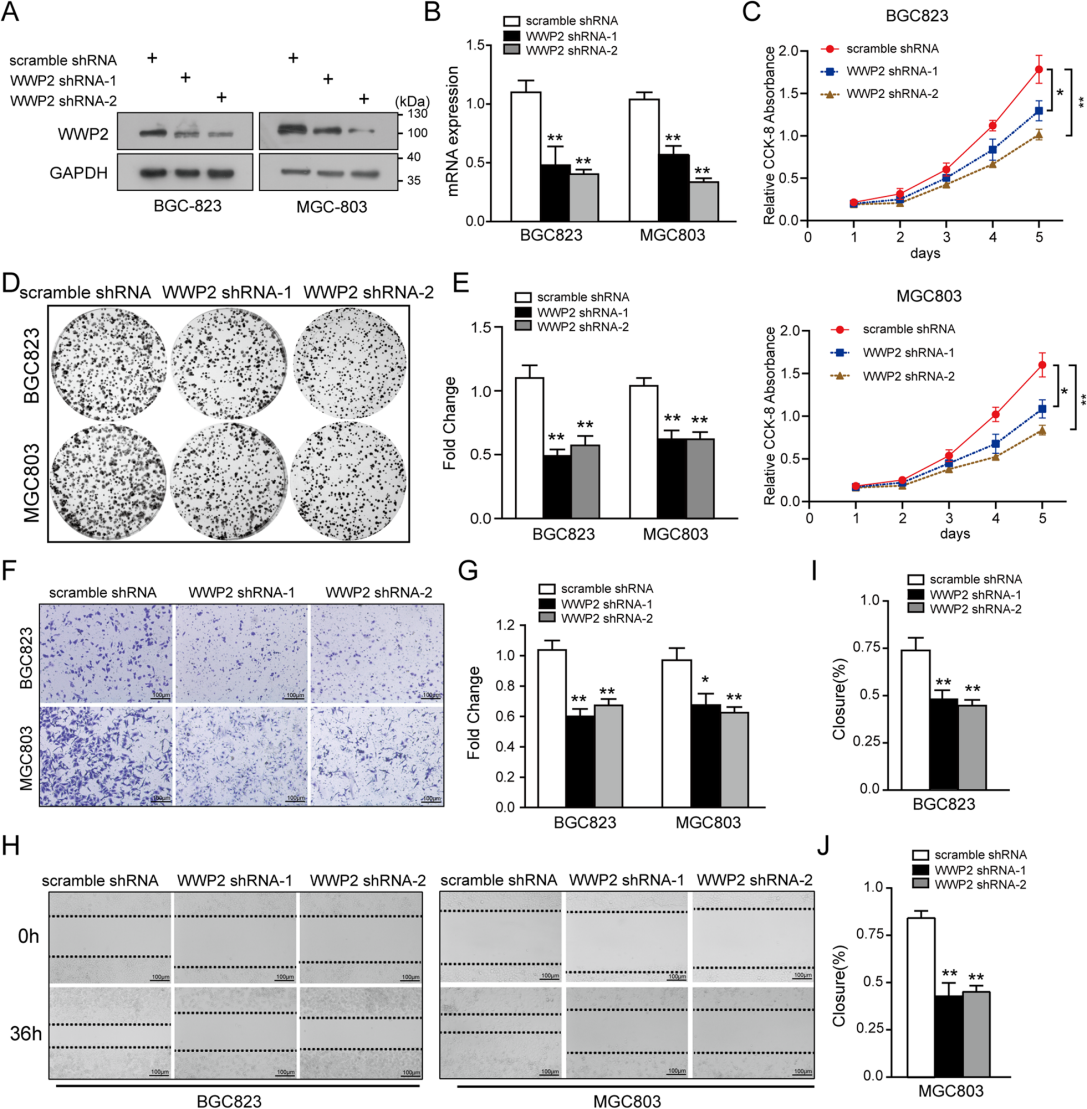
WWP2 knockdown suppresses the aggressive behaviors of GC cells. **A, B** BGC-823 and MGC-803 cells were transfected with WWP2-shRNAs, and the efficiency was validated by western blotting and qRT‒PCR assays. **C** CCK-8 assays were performed to detect cell growth upon WWP2 downregulation. **D, E** Colony formation experiments were used to analyze the effects of WWP2 silencing on cell colony formation. **F**–**J** Transwell invasion and wound healing assays were utilized to assess the invasive and migrative abilities of gastric cancer cells transfected with WWP2-shRNAs or scramble shRNA. One-way ANOVA: *p < 0.05, **p < 0.01

To elucidate the role of WWP2 in GC cells, human WWP2 was overexpressed in HGC-27 and SGC-7901 cells, and WWP2 was downregulated in BGC-823 and MGC-803 cells. We first verified the efficiency of transfection of our WWP2 expression constructs by western blotting and qRT-PCR assays (Figs. 3A–B, 4A–B and Additional file 3: Fig. S1C–D). We found that ectopic expression of WWP2 enhanced the growth and colony formation abilities of HGC-27 and SGC-7901 cells as determined by CCK-8 and colony formation assays (Fig. 3C–E). WWP2 depletion exerted the opposite effects (Fig. 4C–E). Consistent with this observation, WWP2 overexpression enhanced the migration and invasion abilities of GC cells (Fig. 3F–I), while silencing WWP2 induced the opposite effects (Fig. 4F–I). Therefore, these data demonstrate that WWP2 is responsible for GC cancer cell proliferation, migration and invasion in vitro and thus represents a potential therapeutic target for GC patients.

### WWP2 orchestrates the Hippo-YAP1 pathway by ubiquitinating LATS1 protein

**Fig. 5:**
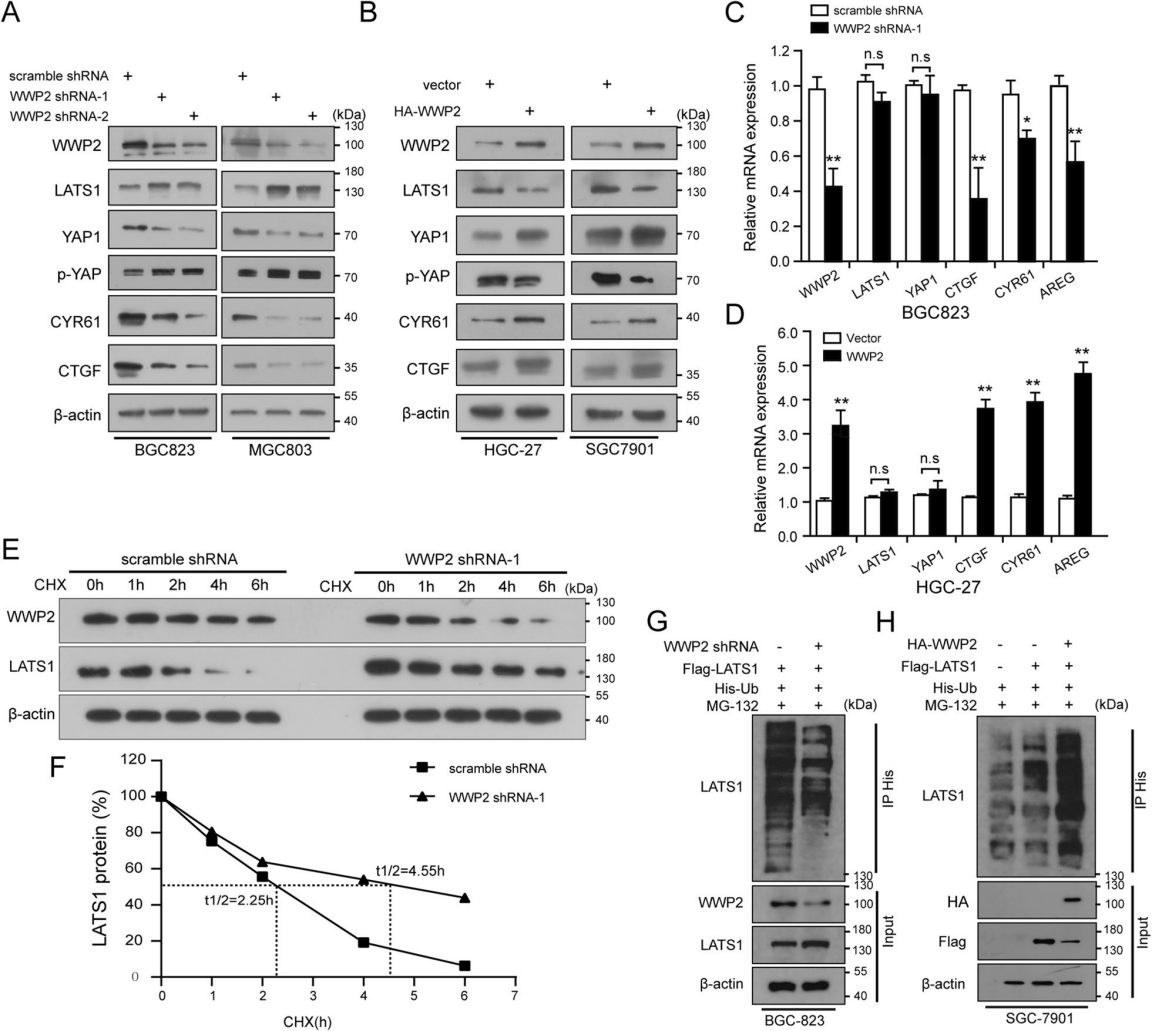
WWP2 orchestrates the Hippo-YAP1 pathway by ubiquitinating LATS1 protein. **A, B** Western blotting assays were performed to detect the protein expression of LATS1/2, YAP1, p-YAP and its downstream target genes CYR61 and CTGF in GC cells upon corresponding treatments as presented. **C, D** qRT‒PCR analysis of the mRNA expression of WWP2, LATS1, YAP1, CTGF, CYR61 and AREG in GC cells upon WWP2 upregulation or knockdown. **E** Western blotting analysis of LATS1 protein in modified SGC-7901 cells upon WWP2 silencing and treatment with CHX (25 μg/mL) for specific time points. **F** The abundance of LATS1 protein was quantified and is shown in line graphs. **G, H** Western blotting analysis of in vivo ubiquitination. **G** BGC-823 cells were co-transfected with WWP2 shRNA or scramble shRNA and treated with MG132 for 6 h before harvesting. **H** SGC-7901 cells were transfected with combinations of plasmids encoding HA-WWP2, Flag-LATS1 and His-Ub, and the cells were treated with MG132 for 6 h before being harvested for further study. Student’s t test: *p < 0.05, **p < 0.01

LATS1 is a main component of the Hippo pathway and inactivates the oncogenic functions of YAP1 through phosphorylation [28]. Given that the coimmunoprecipitation data showed that WWP2 was an interacting partner of LATS1, we speculated that the cancer-promoting functions of WWP2 might be related to the Hippo-YAP1 pathway. As clearly shown in Fig. 5A and Additional file 3: Fig. S1E–F, the protein expression levels of YAP1 and its downstream target genes, CTGF and CYR61, were increased upon WWP2 overexpression, while p-YAP and LATS1 were significantly downregulated. In contrast, WWP2 depletion resulted in opposite expression patterns (Fig. 5B, Additional file 3: Fig. S1G–H). Additionally, as illustrated in Fig. 5C–D, the mRNA levels of the Hippo-YAP1 pathway downstream targets CTGF, CYR61 and AREG were positively correlated with WWP2 levels, but there were no effects on LATS1 mRNA expression, indicating that WWP2 may regulate LATS1 protein expression at the posttranscriptional level.

Given that WWP2 acts as an E3 ligase and could bind to LATS1, we wondered whether LATS1 was regulated and ubiquitinated by WWP2. Therefore, we first assessed whether WWP2 could affect LATS1 protein stability utilizing cycloheximide-chase experiments. In the presence of CHX (protein synthesis inhibitor), we found that WWP2 knockdown significantly prolonged the half-life of the LATS1 protein from 2 to 4 h (*p* < 0.05, Fig. 5E–F), indicating that WWP2 promotes the degradation of LATS1. Many studies have clarified that LATS1 degradation occurs through the proteasome [27, 29, 30], and our results support that WWP2 negatively regulates LATS1 protein stability in a proteasome-dependent manner rather than in a lysosome-dependent manner (Additional file 4: Fig. S2). More interestingly, the in vivo ubiquitination assays revealed that the endogenous ubiquitination of LATS1 protein was markedly suppressed upon WWP2 downregulation in gastric cancer cells (Fig. 5G). In contrast, WWP2 overexpression significantly promoted the ubiquitination of the LATS1 protein (Fig. 5H). Collectively, our data demonstrate that WWP2 regulates the Hippo-YAP1 signaling pathway by accelerating the ubiquitination and degradation of the LATS1 protein.

### LATS1 functions as a crucial mediator of WWP2 to promote GC cell proliferation and invasion

**Fig. 6:**
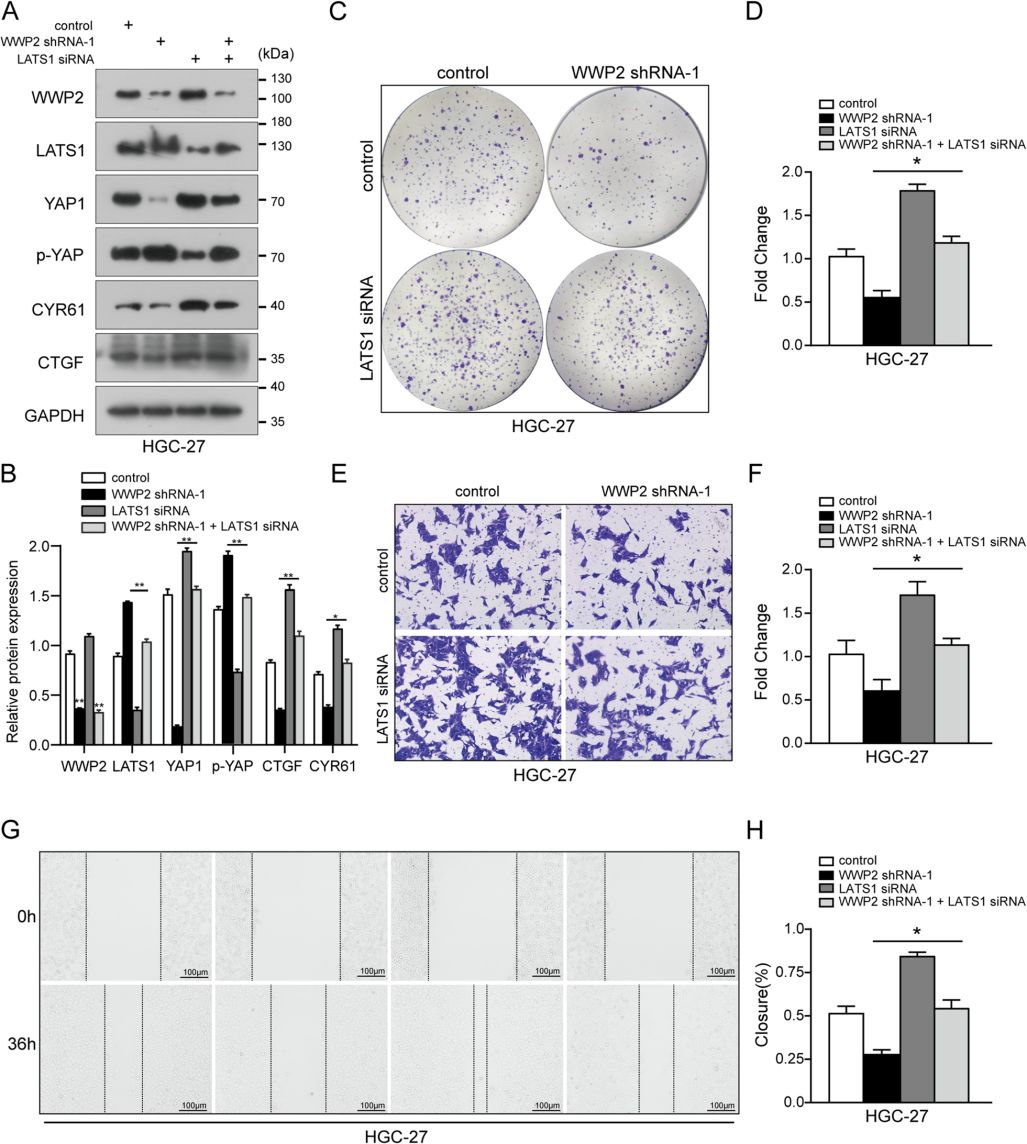
LATS1 functions as a crucial mediator of WWP2 to promote GC cell proliferation and invasion. **A, B** The protein expression of WWP2, LATS1, YAP1, *p*-YAP and its downstream target genes, CTGF and CYR61, was detected by western blotting analysis in WWP2-silenced HGC-27 cells co-transfected with or without LATS1 siRNA. **C, D** Colony formation experiments were performed in WWP2-silenced HGC-27 cells co-transfected with or without LATS1 siRNA. **E–H** The invasive and migrative abilities were analyzed in WWP2-silenced HGC-27 cells co-transfected with or without LATS1 siRNA by transwell **E, F** or wound healing assays **G, H**, respectively. One-way ANOVA: *p < 0.05, **p < 0.01

To further examine whether LATS1 is indispensable for WWP2-mediated GC progression, we manipulated LATS1 and WWP2 expression using siRNA constructs and plasmids in HGC-27 cells and assessed malignant phenotypes. As shown in Fig. 6A, B, WWP2 shRNA reduced the expression of core components of the Hippo signaling pathway, such as YAP1 and its downstream targets, CTGF and CYR61, while LATS1 depletion partially abrogated the effects of WWP2 knockdown. Functional assays further confirmed that the suppression of proliferation, migration and invasion induced by WWP2 silencing was largely rescued by LATS1 knockdown in GC cells (Fig. 6C–H). Furthermore, when LATS1 and WWP2 overexpression plasmids were co-transfected, the overexpression of LATS1 rescued the effects of WWP2 in GC cells (Additional file 5: Fig. S3). Taken together, these data reveal that the LATS1-YAP1 axis is critical for the effects of WWP2 in facilitating the progression of human GC cells.

### WWP2 attenuation impedes the growth of GC cells in vivo

**Fig. 7:**
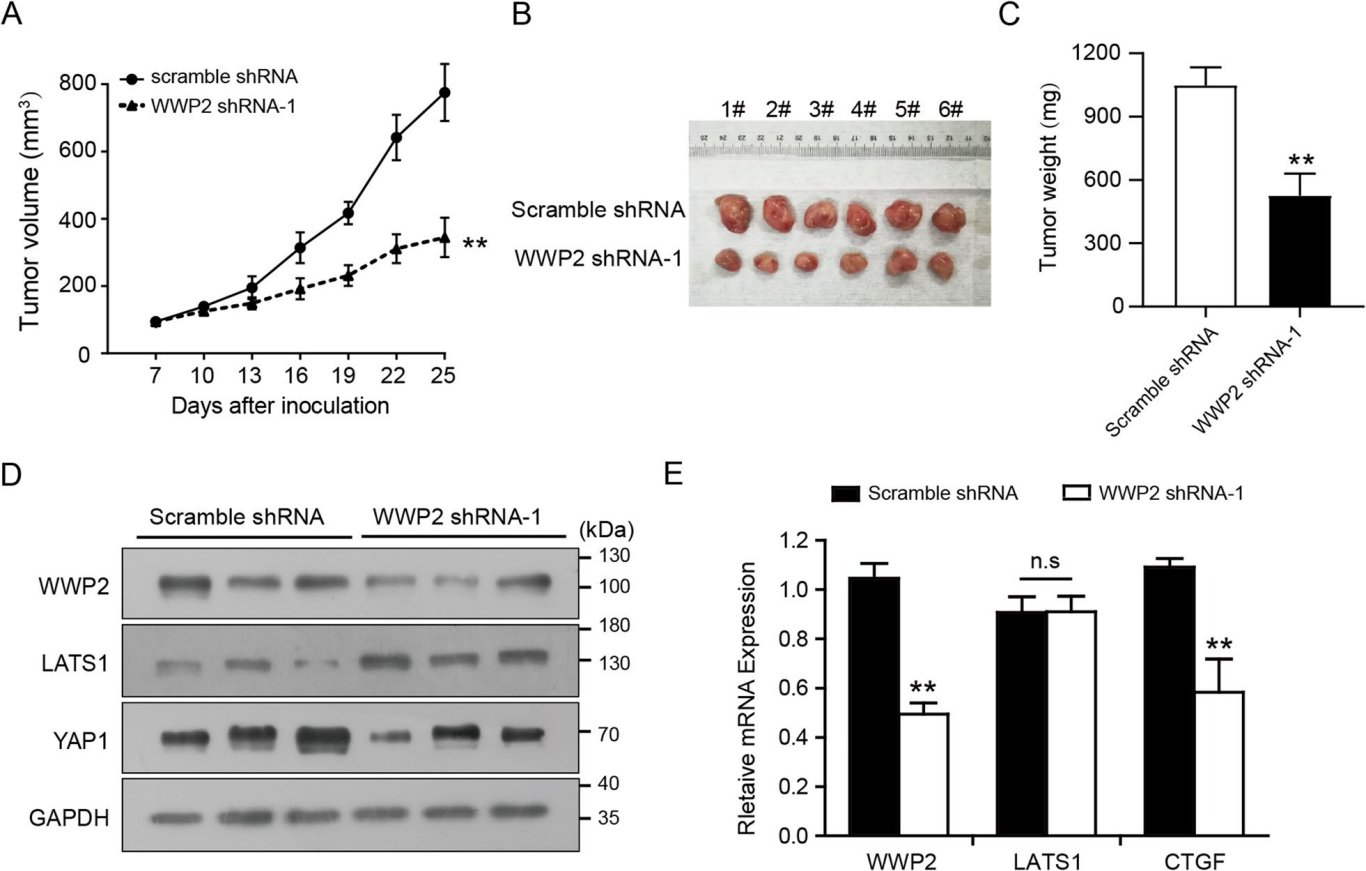
WWP2 attenuation impedes the growth of GC cells in vivo. **A** Growth curves of xenograft tumors extracted from BGC-823 cells transfected with scramble shRNA or WWP2 shRNA-1. **B, C** Images of xenograft tumors were collected, photographed and weighed at the end of the in vivo experiments. **D** The protein expression of WWP2, LATS1 and YAP1 in xenograft tumors was detected utilizing western blotting assays. **E** qRT‒PCR experiments were performed to analyze the mRNA levels of WWP2, LATS1 and CTGF in xenograft tumors. The data are summarized as the mean ± SD of three independent experiments. **P < 0.01 by two-tailed *t* test, n.s indicates no significance

To increase the reliability of our findings and verify our hypothesis, a subcutaneous xenograft nude mouse model was established. GC cells that expressed WWP2 shRNA or scramble shRNA were inoculated into the right armpit of nude mice, and xenograft tumor size was monitored for 25 days. As demonstrated in Fig. 7A, downregulation of WWP2 significantly suppressed the growth of the subcutaneous xenografts. Consistently, WWP2 knockdown resulted in a significant reduction in xenograft tumor volume and weight (Fig. 7B–C). Moreover, the total protein and mRNA of the transplanted tumors were extracted for western blotting and qRT-PCR analysis. In line with the results of the cellular experiments, the protein expression of LATS1 was obviously increased, while the expression of YAP1 was decreased in the WWP2-shRNA groups compared to the scramble groups (Fig. 7D). Consistently, the qRT-PCR data indicated that the expression of LATS1 mRNA was not affected upon WWP2 depletion (Fig. 7E). Collectively, our results reveal that WWP2 contributes to GC progression by modulating the Hippo-YAP1 pathway in vivo.

## Discussion

In this study, we found that WWP2 expression is significantly increased in GC tumor tissues and that overexpression of WWP2 is associated with malignant phenotypes. Mechanistically, WWP2 facilitates the ubiquitination and degradation of the LATS1 protein, thereby mediating the activation of YAP1. Collectively, our findings shed new light on the role and mechanism of WWP2 in GC tumorigenesis and progression.

Aberrant regulation of the Hippo signaling pathway contributes greatly to carcinogenesis and progression owing to its critical roles in controlling organ size and cell proliferation [8]. LATS1 protein is reported to be downregulated in a variety of human tumors, including cervical cancer, lung cancer, breast cancer, colorectal cancer and ovarian cancer [31–35]. In general, LATS1 plays a critical role in orchestrating the Hippo-YAP1 pathway, thereby subsequently leading to the inhibition of proliferation and invasion in cancer cells [4, 36], indicating that targeting LATS1 could be a promising strategy for GC treatment. In our study, for the first time, we identified WWP2 as a novel LATS1-interacting protein by co-IP and immunofluorescence assays. WWP2 was found to be frequently upregulated in human cancers, such as glioma and oral and lung cancers [17, 37, 38]. For instance, WWP2 is overexpressed in liver cancer, and its overexpression promotes cancer growth and metastasis by regulating the PTEN/Akt pathway [21]. Our study suggested that WWP2 was frequently upregulated in GC tissues and that its upregulation was markedly associated with poor clinical outcomes, which was consistent with a recently published study [23]. Moreover, we found that WWP2 facilitated cell proliferation, migration and invasion in GC cells by gain- and loss-of-function assays. In contrast to their study [23], our data uncovered the migration- and invasion-promoting roles of WWP2 in GC cells. Thus, these data, supported by clinical validation studies and cellular experiments, indicate that WWP2 might behave as an oncogene in human GC progression.

An abundance of studies have demonstrated that YAP1 is the core effector of the Hippo pathway. In general, YAP1 expression is mainly controlled by LATS1/2 kinase via phosphorylation [39]. Thus, regulation of LATS1/2 activity is central to mechanisms regulating the Hippo-YAP1 pathway. Recently, quite a few studies have revealed the critical role of proteasomal ubiquitination in the regulation of LATS1 protein. Our and others’ previous data have identified several ubiquitin ligases or modulators, such as CUL4A, SPOP, Itch and NEDD4, which could target LATS1 for ubiquitination, thereby driving tumorigenesis and progression [29, 40–42]. WWP2, as an E3 ubiquitin ligase, was reported to induce oncogenesis and progression by promoting PTEN ubiquitination [13, 43, 44]. Two other members of the NEDD4-like family, NEDD4 and WWP1, are critical for LATS1 protein stability and negatively regulate LATS1 by facilitating its polyubiquitination-mediated degradation [27, 30]. Previously, Tatsuyuki et al. also found that knockdown of WWP2 increased the phosphorylation levels of LATS1 and YAP1 in COS-1 cells [45]; however, the association between WWP2 and LATS1 in carcinogenesis remains unknown. In this study, we first unveiled that WWP2 is a new E3 ligase of the LATS1 protein in GC cells. Specifically, the in vivo ubiquitination and CHX experiments supported that WWP2 regulates the protein stability of the LATS1 protein via the ubiquitin proteasome. Previous studies have shown that LATS1 has two PPxY motifs that can bind to proteins containing the WW domain, such as YAP/TAZ and WWP1 [46–48]. Interestingly, the WWP2 protein also contains four WW domains, which might be involved in the interaction between WWP2 and LATS1. It is worth mentioning that there are also several limitations in our study. Further investigation using GST pull-down assays is required to determine whether WWP2 can directly bind to LATS1 and to determine the precise binding domains between them. Since LATS1 is a tumor suppressor in GC, targeting its upstream regulators, such as WWP2, to increase LATS1 expression could be an ideal therapeutic strategy. Based on our findings and others’, we speculate that WWP2-mediated LATS1 ubiquitination and subsequent YAP1 activation may promote tumor progression, thereby providing novel targets for clinical cancer therapy. It is of great importance to discover or construct small molecular inhibitors of WWP2 in our future study.

Noticeably, the effects of WWP2 downregulation on the downstream target genes of the Hippo pathway and the invasion and proliferation of GC cells could not be completely rescued by LATS1 siRNA, which indicates that LATS1 is not the only effector of WWP2 in GC cells. WWP2 was reported to induce cell proliferation and drug resistance by modulating the PTEN/PI3K/Akt and Notch3 signaling pathways [21, 22]. These lines of evidence indicate that WWP2 may also interact with these various genes and pathways in GC.

**Fig. 8:**
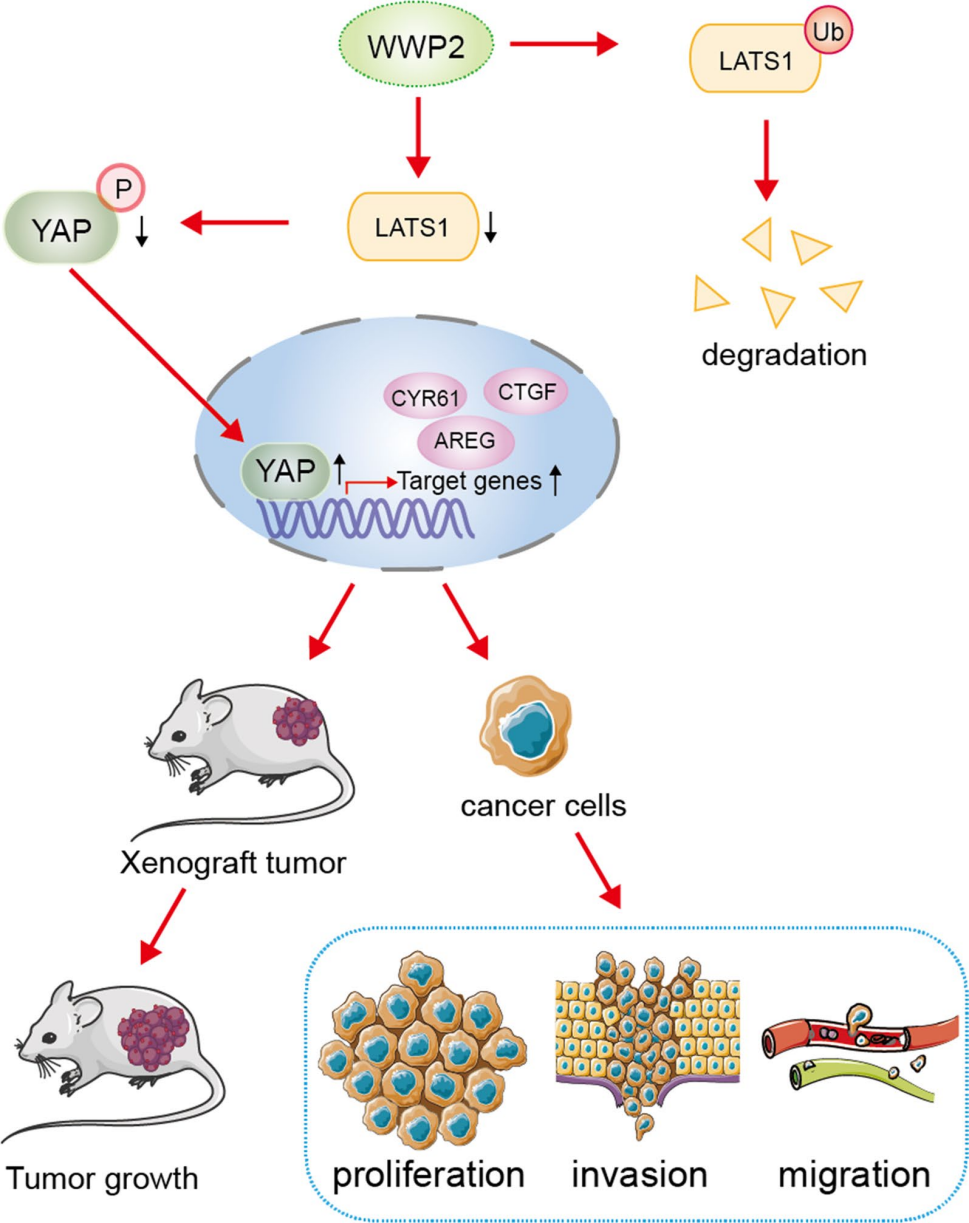
Schematic diagram depicting the mechanism of WWP2-mediated GC progression via LATS1 ubiquitination and subsequent destruction. WWP2 upregulation contributes to the promotion of cell proliferation, migration and invasion of GC cells. WWP2 triggers GC tumorigenesis and progression by facilitating LATS1 ubiquitination and degradation, thereby inducing YAP1 activation

Conclusively, our data provided new insights into the molecular mechanisms of WWP2-driven GC progression, by which WWP2 facilitates the ubiquitination and degradation of the LAST1 protein (Fig. 8).

## Supplementary Information

**Additional file 1: Table S1: **The clinicopathological characteristics of gastric cancer patients

**Additional file 2: Table S2: **The sequences of WWP2 shRNAs and LATS1 siRNAs used in this study

**Additional file 3: Fig. S1: **Supplemental qRT-PCR analysis results and quantitative western blotting results

**Additional file 4: Fig. S2: **WWP2 negatively regulates LATS1 stability in a proteasome-dependent manner

**Additional file 5: Fig. S3: **LATS1 rescues the oncogenic effects of WWP2 overexpression

## Data Availability

The datasets used and analyzed during the current study are available from the corresponding authors (Xiaojun Xiang and Ziling Fang) upon reasonable request.

## Abbreviations

WWP2: WW domain-containing E3 ubiquitin ligase 2
LATS1: Large tumor suppressor kinase 1
GC: Gastric cancer
CTGF: Connective tissue growth factor
CYR61: Cysteine-rich angiogenic inducer 61
Co-IP: Coimmunoprecipitation
IHC: Immunohistochemistry
CHX: Cycloheximide
