# Supplementary material for: WWP2 drives the progression of gastric cancer by facilitating the ubiquitination and degradation of LATS1 protein
Source: Cell Commun Signal. 2023 Feb 17;21:38. doi: 10.1186/s12964-023-01050-2 (PMC9938551; doi:10.1186/s12964-023-01050-2)
Supplement: Supplementary file 2 — Additional file 1: Table S1: The clinicopathological characteristics of gastric cancer patients [file 12964_2023_1050_MOESM2_ESM.docx]

**Supplementary Table S1: The clinicopathological characteristics of gastric cancer patients.**

| **Patients** | **Age (year)** | **gender** | **Tumor size (cm)** | **Depth of tumor invasion** | **Differentiation**  **(well, moderate and poorly)** | **TNM stage** | **Lymph node metastasis**  **(N_0_ or N_X_)** |
| --- | --- | --- | --- | --- | --- | --- | --- |
| #1 | 62 | Male | 6*4*1.5 | T_4_ | moderate | III | N_X_ |
| #2 | 69 | Male | 3*2.9*1.8 | T_3_ | moderate | III | N_X_ |
| #3 | 64 | Male | 2.8*2.2*0.8 | T_4_ | poorly | III | N_X_ |
| #4 | 54 | Female | 4*4*1.3 | T_2_ | well | II | N_X_ |
| #5 | 56 | Male | 3*3*1 | T_4_ | poorly | III | N_X_ |
| #6 | 92 | Male | 3.5*4*2.5 | T_4_ | moderate | III | N_X_ |
| #7 | 55 | Male | 2.5*2*0.3 | T_2_ | moderate | II | N_X_ |
| #8 | 55 | Male | 3.2*2.7*1.5 | T_4_ | moderate | III | N_X_ |
