# Supplementary material for: WWP2 drives the progression of gastric cancer by facilitating the ubiquitination and degradation of LATS1 protein
Source: Cell Commun Signal. 2023 Feb 17;21:38. doi: 10.1186/s12964-023-01050-2 (PMC9938551; doi:10.1186/s12964-023-01050-2)
Supplement: Supplementary file 3 — Additional file 2: Table S2: The sequences of WWP2 shRNAs and LATS1 siRNAs used in this study [file 12964_2023_1050_MOESM3_ESM.docx]

**Supplementary Table S2: The sequences of WWP2 shRNAs and LATS1 siRNAs used in this study.**

| **Gene** | **Sequences for shRNAs and siRNA** |
| --- | --- |
| WWP2 shRNA-1 | 5′-CACCTCACCTACTTCCGCTTT -3′ |
| WWP2 shRNA-2 | 5′-TGGCAGAAGAACGCCATCTAT -3′ |
| LATS1 siRNA | 5’ -GGUAGUUCGUCUAUAUUAUTT -3’ |
