# Supplementary figures and images for: WWP2 drives the progression of gastric cancer by facilitating the ubiquitination and degradation of LATS1 protein
Source: Cell Commun Signal. 2023 Feb 17;21:38. doi: 10.1186/s12964-023-01050-2 (PMC9938551; doi:10.1186/s12964-023-01050-2)

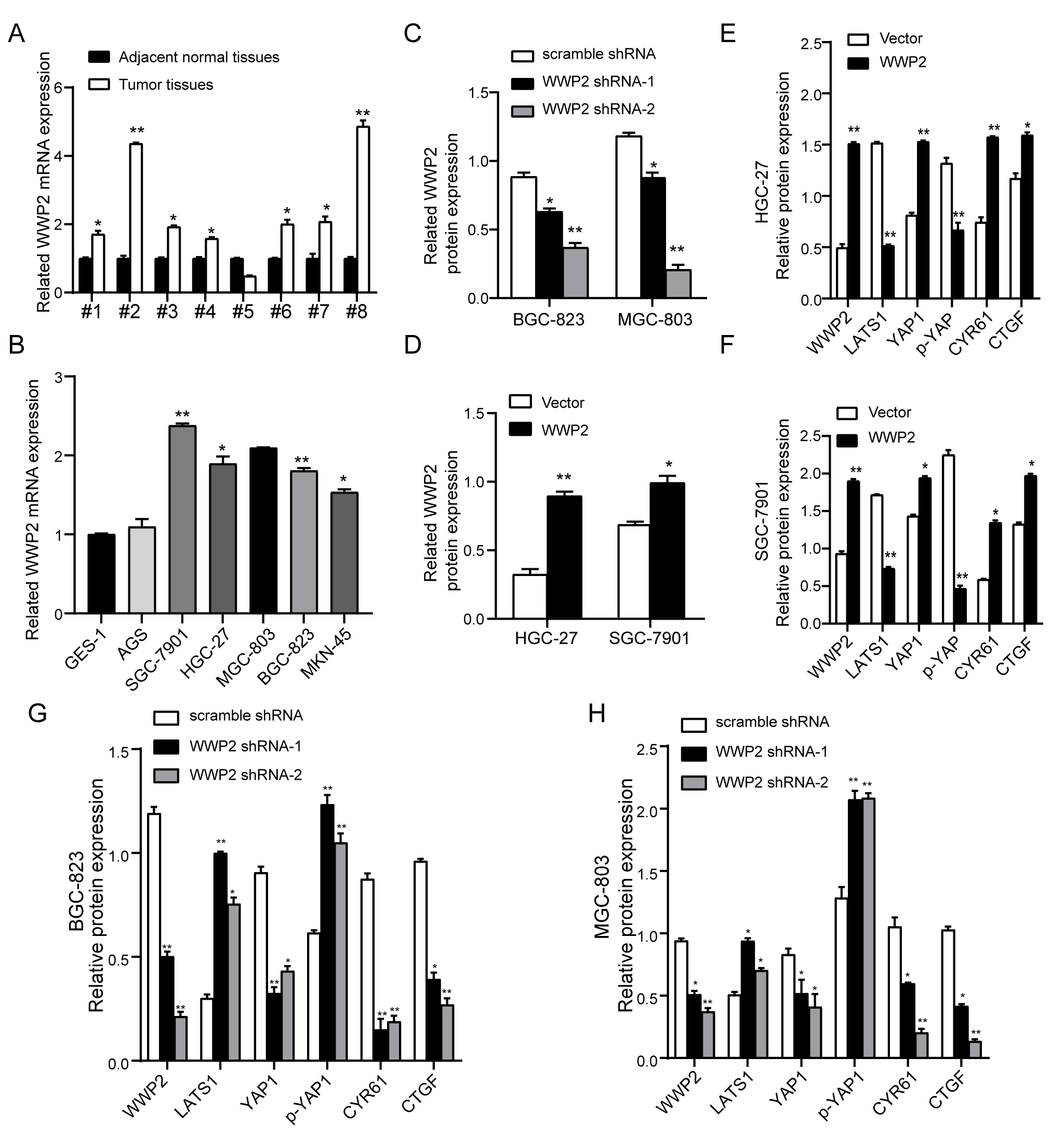

Supplement: Supplementary file 4 — Additional file 3: Fig. S1: Supplemental qRT-PCR analysis results and quantitative western blotting results [file 12964_2023_1050_MOESM4_ESM.tif]

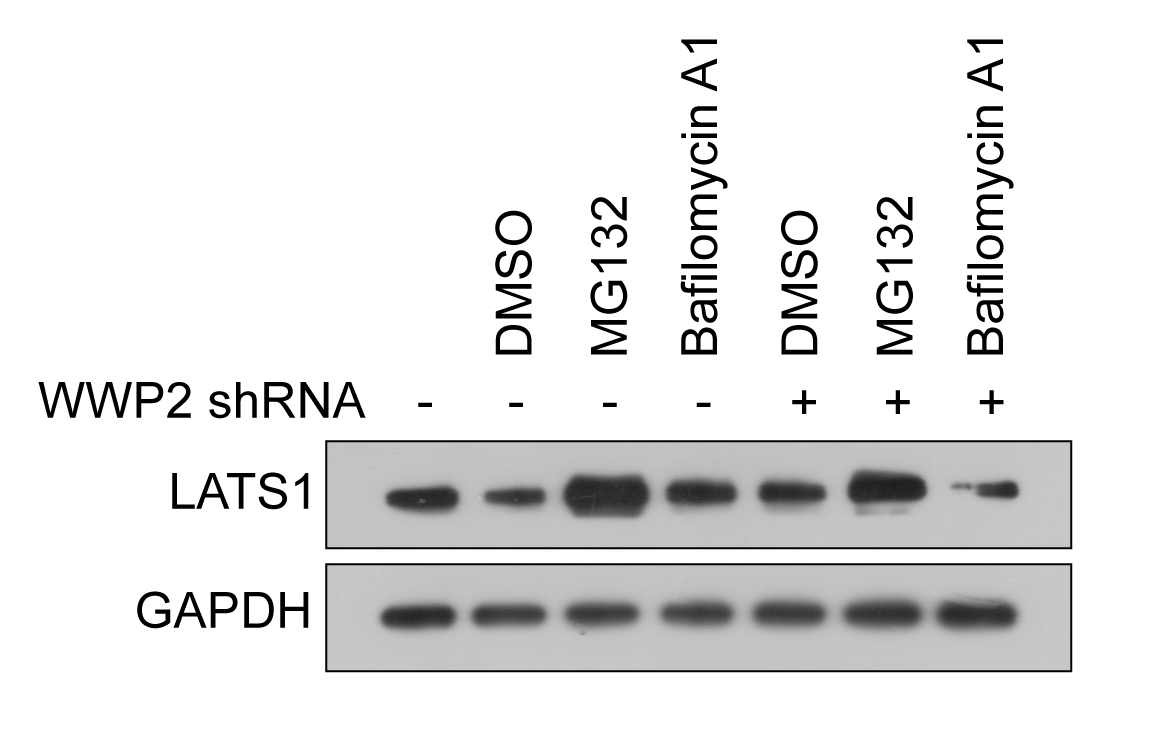

Supplement: Supplementary file 5 — Additional file 4: Fig. S2: WWP2 negatively regulates LATS1 stability in a proteasome-dependent manner [file 12964_2023_1050_MOESM5_ESM.tif]

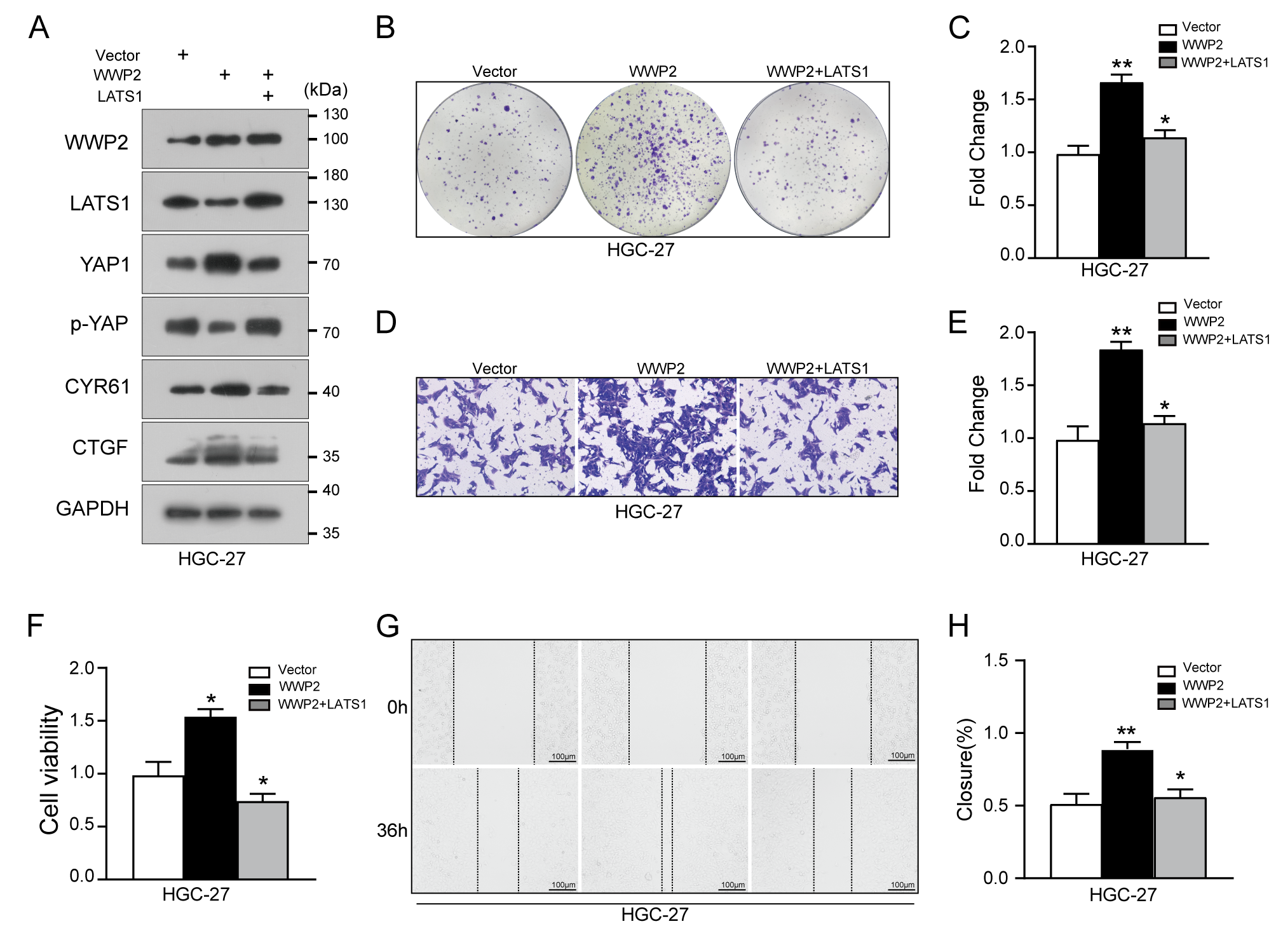

Supplement: Supplementary file 6 — Additional file 5: Fig. S3: LATS1 rescues the oncogenic effects of WWP2 overexpression [file 12964_2023_1050_MOESM6_ESM.tif]
